# Detailed Anatomy of the Lateral Rectus Muscle-Superior Rectus Muscle Band

**DOI:** 10.1155/2019/5374628

**Published:** 2019-07-10

**Authors:** Yong Seok Nam, Yooyeon Park, In-Beom Kim, Sun Young Shin

**Affiliations:** ^1^Department of Anatomy and Institute for Applied Anatomy, Seoul St. Mary's Hospital, College of Medicine, The Catholic University of Korea, Seoul, Republic of Korea; ^2^Department of Ophthalmology and Visual Science, Seoul St. Mary's Hospital, College of Medicine, The Catholic University of Korea, Seoul, Republic of Korea

## Abstract

**Background:**

To elucidate the detailed anatomy of the lateral rectus muscle-superior rectus muscle (LR-SR) band by cadaver eye investigation.

**Methods:**

In total, 48 eyes of 24 cadavers were observed macroscopically. The lateral rectus muscle (LRM) and superior rectus muscle (SRM) were dissected from their origins to their insertions under the dissecting microscope, and the location, width, length, and tensile strength of the LR-SR bands were measured.

**Results:**

The LR-SR band is the thick ligament interconnecting LRM pulleys with SRM pulleys. The LR-SR band was covered by the orbital part of the lacrimal gland superiorly, and the intermuscular septum between the LRM and SRM was shown anterior to the LR-SR band. The length of the attachment site between the LR-SR band and the SRM was less at 8.64 ± 1.52 mm (*p*=0.040), its thickness was thinner at 0.74 ± 0.16 mm (*p*=0.002), and its tensile strength was weaker at 7.64 ± 1.82 N (*p*=0.028) compared to the attachment site between the LR-SR band and the LRM.

**Conclusions:**

This study revealed the detailed anatomy of the LR-SR band and provided helpful information to understand heavy eye syndrome and sagging eye syndrome.

## 1. Background

Orbital connective tissue is an important component that closely cooperates with the extraocular muscles [1–8]. Each rectus muscle is encircled by a pulley, a ring of primarily collagenous connective tissue located posterior to the globe equator and contiguous with Tenon's fascia. Previous histological studies identified suspensory bands of the connective tissue [1–5]. A dense band is present from the medial rectus muscle to the inferior rectus muscle pulleys and from the medial rectus muscle to the superior rectus muscle pulleys. Another dense band is present from the lateral rectus muscle to superior rectus muscle pulleys (LR-SR band) [4]. It is well known that orbital connective tissue degenerates with aging, which could be one of the causes of adult-onset strabismus, and the age-related degeneration might influence on the band between the rectus muscles [9–12].

Heavy eye syndrome affects elderly patients with high myopia. There are progressive esotropia and often hypotropia with limited abduction and supraduction in heavy eye syndrome patients [13–15]. The anatomical hallmark of heavy eye syndrome is superotemporal prolapse of the myopic globe. Recently, a similar condition in nonmyopic elderly patients, sagging eye syndrome, has been reported to be associated with age-related orbital connective tissue degeneration, involving external manifestations of baggy eyelids, superior sulcus deformities, aponeurotic blepharoptosis, and the likelihood of previous blepharoplasty [7, 10, 12]. In both heavy eye syndrome and sagging eye syndrome, the LR-SR bands are reported to be either elongated or ruptured [12]. It is clear that degeneration of the LR-SR band contributes to the pathology of both conditions [7, 10, 12].

Thus, the anatomy of the LR-SR band and its relationships to the superior rectus muscle (SRM) and the lateral rectus muscle (LRM) is of great significance. However, limited information is known up to date on the detailed anatomy of the LR-SR band. This prompted us to investigate the detailed anatomy of this band using cadavers.

## 2. Materials and Methods

Twenty-four registered cadavers were approved with appropriate consent from the Department of Anatomy and the Institute for Applied Anatomy at the Catholic University of Korea. The mean age of death was 73.6 ± 12.8 years (range 42∼91 years). The methods for securing human tissue adhered to the tenets of the Declaration of Helsinki.

Forty-eight intact eyes extracted from 24 fresh cadavers (with no formaldehyde preservative) without having been frozen previously were involved in the study. The medical records of the subjects showed no prior history of eye muscle or orbital disorders, eyelid, eye muscle surgeries, and trauma. The most common causes of death were cardiac and respiratory-related diseases, followed by neoplastic processes.

Preparation and dissection were performed as we previously described [13]. An oscillating saw was used in the process of making a transverse incision 2 cm superior to the upper orbital rim and 2 cm inferior to the lower orbital rim. Taking care to preserve the periorbital and orbital apexes, a rongeur was used in the process of removing the orbital floor.

**Figure 1:**
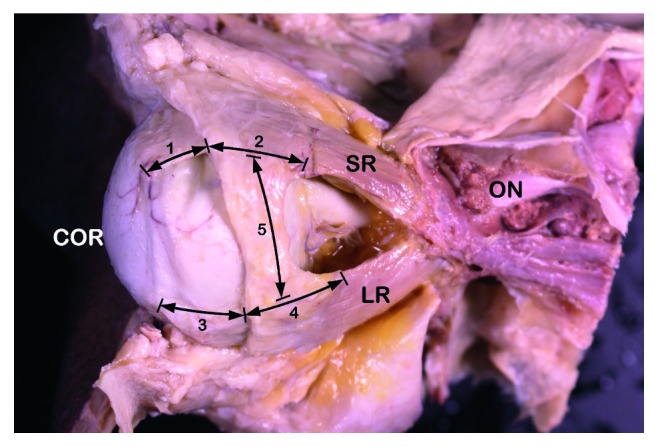
The orbit illustrating definitions of anatomical distances. (1): the distance from the anterior border of the lateral rectus muscle-superior rectus muscle (LR-SR) band to the SR insertion; (2): the length of the attachment site between the LR-SR band and the SR: (3), the distance from the anterior border of the LR-SR band to the LR insertion; (4): the length of the attachment site between the LR-SR band and the LR; (5): the width of the LR-SR band. ON = optic nerve; LR = lateral rectus muscle; SR = superior rectus muscle.

The orbital fat tissue and the fascia were dissected microanatomically with accuracy from the origin of the SRM and the LRM to the insertion, using a dissection microscope. After the orbital fat of the lacrimal gland and fat were carefully removed, the relationships between the LRM, SRM, and LR-SR bands were characterized. The LR-SR band of each specimen was photographed, and the width and length of the LR-SR band were determined using a digimatic caliper (CD-15CP, Mitutoyo, Kawasaki, Japan). The lengths of the attachment site between the LR-SR band and the SRM or the LRM were measured (Figure 1). Finally, the tensile strength of the attachment site between the LR-SR band and the LRM or the SRM was determined using a force gauge (BFG 200 N, Mecmesin, Slinfold, UK). A loop of 5–0 black silk was carefully introduced through the experimental sites using a half round cutting needle. Three-centimeter-long thread loop was pulled using a tensiometer. The breaking strength was measured.

Statistical analyses were performed using SPSS for Windows software (ver. 25.0; SPSS Inc., Chicago, IL, USA). For all tests, significance was set at a value of *p* < 0.05. Differences in anatomic variables were compared using *t* tests as appropriate.

## 3. Results

**Figure 2:**
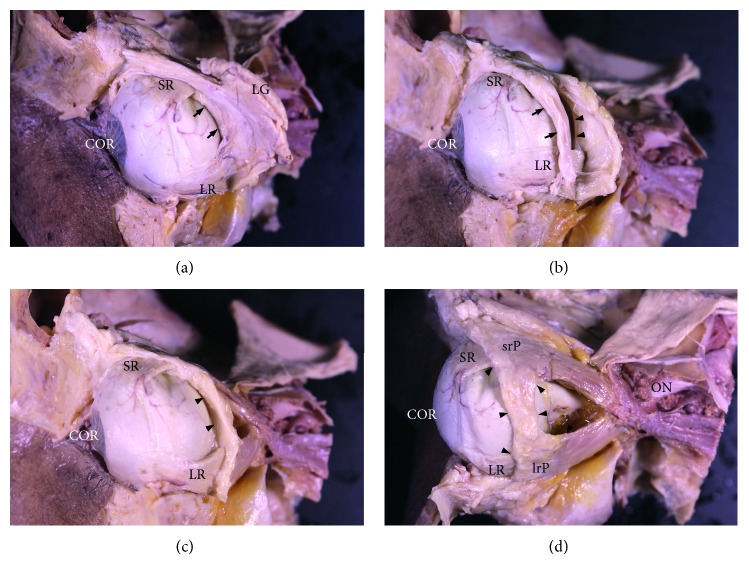
The anatomy of the lateral rectus muscle-superior rectus muscle (LR-SR) band and its relationship with adjacent structures. (a) The orbital part of the lacrimal gland (LG) covers the LR-SR band, and the intermuscular septum is shown anterior to the LR-SR band. (b) After excising the orbital part of the LG, the anterior border of LR-SR band is revealed. (c, d) After excising the intermuscular septum, the whole LR-SR band is revealed and connected lateral rectus and superior rectus pulleys. Arrows indicate the intermuscular septum between the LR and SR. Arrowheads indicate the LR-SR band. ON = optic nerve; srP = superior rectus pulley; irP = inferior rectus pulley.

The LR-SR band was the ligament interconnecting the LRM and SRM pulleys.  Figure 2 shows the anatomical relationships of the LR-SR band with adjacent structures. The LR-SR band was covered by the orbital part of the lacrimal gland superiorly, and the intermuscular septum between the LRM and SRM was shown anterior to the LR-SR band. After excising the orbital part of the lacrimal gland, the anterior border of LR-SR band was revealed. After excising the intermuscular septum, the LR-SR band was revealed and connected the LRM and SRM pulleys.

The anterior border of the LR-SR band was located 9.04 ± 1.53 mm from the insertion of the SRM and 9.80 ± 1.54 mm from the insertion of the LRM. The width of the LR-SR band was 11.82 ± 1.63 mm at the anterior border. Its thickness was 0.83 ± 0.12 mm in the middle, 0.95 ± 0.24 mm at the side attached to the LRM, and 0.74 ± 0.16 mm at the side attached to the SRM. The thickness of the side attached to the LRM was greater than the thickness of the side attached to the SRM (*p*=0.002). The length of the attachment between the LR-SR band and the LRM was 9.62 ± 1.47 mm, and the length of the attachment between the LR-SR band and the SRM was 8.64 ± 1.52 mm, which was less than that of the attachment between the LR-SR band and the LRM (*p*=0.040). The tensile strength of the portions attached to the LRM and the SRM was 9.84 ± 1.57 N and 7.64 ± 1.82 N, respectively. There was a significant difference between these two values (*p*=0.028).

## 4. Discussion

The current study shows that the LR-SR band was the thick ligament interconnecting the SRM pulley with the LRM pulley, which spread horizontally and attached to both the surface and the undersurface of the SRM and LRM. The attachment site between the LR-SR band and the SRM was thinner and weaker compared to the attachment site between the LR-SR band and the LRM.

Kono et al. described the suspensory bands of connective tissue between the rectus muscles in their histologic study and reported the band thickness was lower in the fetal and 93-year-old specimens than in the mid-aged specimens [4]. The LR-SR band had lesser collagen in the 93-year-old specimens than in the mid-aged specimens.

A previous study measured the length of the LR-SR band by digital tracing of MRI and reported that its length was approximately 12.4 mm in older and 8.5 mm in younger controls [7]. In younger controls, the LR-SR band could be visualized as far as 2.9 mm posterior to the globe-optic nerve junction, compared with 3.8 mm in older controls. In this study, the length of the attachment between the LR-SR band and the LRM was 9.62 ± 1.47 mm and the length of the attachment between the LR-SR band and the SRM was 8.64 ± 1.52 mm. This discrepancy may be caused by differences in age and ethnicity of subjects. This study classified 69.4-year-old subjects as the older controls and 23-year-old subjects as the younger controls. We could not obtain cadaveric orbits less than 40 years of age. In addition, the average age at the time of death of our subjects was 76.8 years, which was greater than that of the older controls in the previous study. In addition, there are differences in the measuring method and location.

The MRI finding of sagging eye syndrome and heavy eye syndrome was that the LR-SR band is thinner, elongating, and frequently ruptured [12]. MRI evidence was superotemporal bowing of the band in milder sagging eye syndrome cases and abrupt termination of an attenuated LR-SR band remnant in the superolateral orbit in more severe cases [7]. We could not obtain the cadaveric orbits of sagging eye syndrome and heavy eye syndrome patients and did not reveal the gross anatomy of the LR-SR band in sagging eye syndrome and heavy eye syndrome patients in this study.

Interestingly, our results suggest reasons why the dehiscence of the LR-SR band occurs at the attachment sites between the LR-SR band and the SRM in sagging eye syndrome. The attachment between the LR-SR band and the SRM was weaker than the attachment between the LR-SR band and the LRM. In addition, age-related degeneration made the attachment sites between the LR-SR band and the SRM much weaker. Aging changes of the LRM and SRM pulleys might affect the weakness of the LR-SR band. These would cause the dehiscence of the LR-SR band at the attachment site between the LR-SR band and the SRM.

The anatomy findings of the LR-SR band in our study recommend eye surgeons performing LR-SR band repairs to suture with 8-9 mm in the LR-SR band and the SRM and 9-10 mm away from the SRM insertion.

Our study has some limitations. First, we could not obtain a lot of cadavers with various ages at time of death. Second, we investigated Korean cadaveric eyes; hence, there would be some differences from the previous publications, which included Caucasian cadavers and living MRI volunteers. Third, the connective tissue degeneration after death may influence the parameters of the LR-SR band. In addition, we could not obtain any information of refractive error such as high myopia.

## 5. Conclusions

Our study provides important information for understanding the role of the LR-SR band with sagging eye syndrome and heavy eye syndrome. The results confirm that the attachment between the LR-SR band and the SRM is thinner and weaker compared to the one between the LR-SR band and the LRM. This may be one of the causes for detachment of the LR-SR band at the SRM side.

Further study based on high resolution MRI of patients with and without strabismus will be needed to get further conclusions.

## Data Availability

The datasets generated and/or analysed during the current study are available from the corresponding author on reasonable request.

## Abbreviations

LR-SR band: Lateral rectus muscle-superior rectus muscle band
LRM: Lateral rectus muscle
SRM: Superior rectus muscle.
